# Impact of Regional Left Ventricular Function on Outcome for Patients with AL Amyloidosis

**DOI:** 10.1371/journal.pone.0056923

**Published:** 2013-03-08

**Authors:** Dan Liu, Kai Hu, Markus Niemann, Sebastian Herrmann, Maja Cikes, Stefan Störk, Meinrad Beer, Philipp Daniel Gaudron, Caroline Morbach, Stefan Knop, Eva Geissinger, Georg Ertl, Bart Bijnens, Frank Weidemann

**Affiliations:** 1 Department of Internal Medicine I, University of Würzburg, Würzburg, Germany; 2 Comprehensive Heart Failure Center, University of Würzburg, Würzburg, Germany; 3 Department of Cardiovascular Diseases, University Hospital Center Zagreb and University of Zagreb School of Medicine, Zagreb, Croatia; 4 Department of Radiology, University of Würzburg, Würzburg, Germany; 5 Department of Internal Medicine II, University of Würzburg, Würzburg, Germany; 6 Institute of Pathology, University of Würzburg, Würzburg, Germany; 7 ICREA - Universitat Pompeu Fabra, Barcelona, Spain; 8 Department of Cardiovascular Diseases, K.U. Leuven, Leuven, Belgium; University Hospital Düsseldorf, Germany

## Abstract

**Objectives:**

The aim of this study was to explore the left ventricular (LV) deformation changes and the potential impact of deformation on outcome in patients with proven light-chain (AL) amyloidosis and LV hypertrophy.

**Background:**

Cardiac involvement in AL amyloidosis patients is associated with poor outcome. Detecting regional cardiac function by advanced non-invasive techniques might be favorable for predicting outcome.

**Methods:**

LV longitudinal, circumferential and radial peak systolic strains (Ssys) were assessed by speckle tracking imaging (STI) in 44 biopsy-proven systemic AL amyloidosis patients with LV hypertrophy (CA) and in 30 normal controls. Patients were divided into compensated (n = 18) and decompensated (n = 26) group based on clinical assessment and followed-up for a median period of 345 days.

**Results:**

Ejection fraction (EF) was preserved while longitudinal Ssys (LSsys) was significantly reduced in both compensated and decompensated groups. Survival was significantly reduced in decompensated group (35% vs. compensated 78%, *P* = 0.001). LSsys were similar in apical segments and significantly reduced in basal segments between two patient groups. LSsys at mid-segments were significantly reduced in all LV walls of decompensated group. Patients were further divided into 4 subgroups according to the presence or absence of reduced LSsys in no (normal), only basal (mild), basal and mid (intermediate) and all segments of the septum (severe). This staging revealed continuously worse prognosis in proportion to increasing number of segments with reduced LSsys (mortality: normal 14%, mild 27%, intermediate 67%, and severe 64%). Mid-septum LSsys<11% suggested a 4.8-fold mortality risk than mid-septum LSsys≥11%. Multivariate regression analysis showed NYHA class and mid-septum LSsys were independent predictors for survival.

**Conclusions:**

Reduced deformation at mid-septum is associated with worse prognosis in systemic amyloidosis patients with LV hypertrophy.

## Introduction

Cardiac involvement in patients with light-chain (AL) amyloidosis is responsible for more than 50% of all amyloidosis related deaths, and the median survival time is<6 months in untreated patients with congestive heart failure [1], [2]. LV wall thickening is a common finding indicating cardiac involvement in patients with AL amyloidosis and the degree of hypertrophy is positively related to poor outcome in these patients [3]. Typical conventional echocardiographic features of cardiac involvement in AL amyloidosis patients include increased left and right ventricular wall thickness, normal or small left ventricular (LV) cavity, sparkling texture of myocardium, enlarged left and right atria, pericardial effusion, and advanced diastolic dysfunction [4]–[6]. Two-dimensional speckle tracking imaging (STI) has recently emerged as a method for non-invasive detection of regional myocardial dysfunction, and allows the diagnosis and treatment of cardiac dysfunction in cardiovascular disease [7]–[9]. The aim of this study was to compare the deformation changes in compensated and decompensated biopsy-proven AL amyloidosis patients with LV hypertrophy (CA) and to explore the impact of myocardial deformation changes on clinical staging and outcome in these patients. Our hypothesis was that the evaluation of deformation changes in patients with CA is superior to the degree of hypertrophy as well as left ventricular ejection fraction (EF) for predicting prognosis in these patients.

## Methods

### Ethics Statement

Written informed consent was obtained from all patients or their guardians. The study was approved by Local Ethics Committee at the University of Würzburg and conducted in accordance to the Declaration of Helsinki.

### Study Population and Study Protocol

After excluding patients with coronary artery disease, moderate to severe cardiac valve stenosis, moderate to severe hypertension, and hypertrophic cardiomyopathies unrelated to amyloidosis, 60 consecutive biopsy-proven patients with AL amyloidosis, hospitalized between January 2005 and April 2011 in the university hospitals of Würzburg (n = 55) and Zagreb (n = 5), were screened for initial analysis. At least one biopsy specimen from endomyocardial tissue, bone marrow, rectum, kidney, or subcutaneous fat was positive for amyloid. The presence of amyloid was visualized by Congo red staining, producing apple-green birefringence under polarized light. The plasma cell disorder was assessed by immunohistochemical staining of the bone marrow for κ and λ light chains, and by serum/urine Ig and free light chain testing. Organ systemic involvement was defined by clinical and laboratory manifestations of renal, cardiac, hepatic, gastrointestinal, neuropathic, pulmonary, or soft tissue involvement according to recently published consensus criteria by specialists in cardiology and haematology [10]. Haematological response to treatment was defined as a≥50% decrease in serum and urine monoclonal component [11]. The response was evaluated every 3 months by monitoring serum and urine level of monoclonal protein.

Sixteen out of 60 systemic amyloidosis patients were excluded because of the lack of LV hypertrophy (LV mean thickness <12 mm) during echocardiography examination. The remaining 44 patients with increased LV thickness (LV mean thickness ≥12 mm), defined as cardiac amyloidosis, were included for final analysis. Decompensated CA patients were defined as New York Heart Association (NYHA) functional class >2 and exacerbation of dyspnea within the last 6 months [12]. Thirty healthy volunteers recruited from the local hospital staff matched with age and gender to the patient cohort served as controls. Patients were followed up by clinical visit or telephone call for a median of 345 days (quartiles: 141–846 days).

### Standard Echocardiographic Measurements

A standard echocardiographic examination was performed (GE Vingmed Vivid 7, Horten, Norway). Left ventricular end-diastolic (LVEDD), end-systolic dimensions (LVESD) end-diastolic thickness of the posterior wall (LVPWd) and the septum (IVSd), LV stroke volume (SV), and fractional shortening (FS) were measured using standard M-mode in parasternal LV long axis views. Left atrial (LA) end-systolic diameter (LAD) was measured with 2D mode from the parasternal long-axis view. LV mean thickness was calculated as: (LVPWd+IVSd)/2. From the apical 4-chamber view, right ventricular end-diastolic dimension (RVEDD) and right ventricular (RV) free wall end-diastolic maximal thickness (RVd), end-systolic right atrium area (RAA) and end-systolic interatrial septum maximal thickness (IASd) were measured.

LV EF was measured with the biplane Simpson method in apical 4- and 2-chamber views, septal and lateral mitral annular displacement (MAD_sept and MAD_lat) and tricuspid plane annular systolic excursion (TAPSE) were measured by M-mode in apical 4-chamber view.

Pulsed-wave Doppler was performed in the apical 4-chamber view to obtain mitral inflow velocities for LV filling pattern evaluation. Diastolic function was graded according to recent guidelines [13] and not graded in patients with atrial fibrillation.

### Speckle Tracking Imaging

Deformation was analyzed off-line using EchoPAC software (GE, Horten, Norway). All of the 2D grey scale images were recorded with a frame rate of 40 to 80 frames per second and care was taken to ensure that the entire ventricular wall was clearly visible in all frames. A region of interest (ROI) was created by manually outlining the endocardial border on the apical 4-, 2-chamber, or long-axis views at end-systolic frame. Thereafter, the system automatically tracked the tissue within the region and divided the myocardium into standard segments. The tracking was visually checked and, if necessary, adjusted. The trace analysis was automatically displayed after validating the tracking. Longitudinal peak systolic strain rate (LSRsys) and strain (LSsys) were extracted from basal, mid, and apical segments in LV 6 walls (septum, lateral, inferior, anterior, posterior and anteroseptal wall). Global LSRsys and LSsys for all segments of each wall were obtained by averaging strain rate and strain values from apical 4-, 2-chamber and long-axis views. Circumferential (CSsys) and radial peak systolic strain (RSsys) were detected from short axis views of the LV at papillary muscle level.

### Cardiac Magnetic Resonance Imaging

Cardiac magnetic resonance imaging (cMRI) was performed with a 1.5 Tesla scanner (Magnetom Symphony Quantum, Siemens) or a 3 Tesla scanner (Magnetom Trio, Siemens), using two conventional six-channel body phased-array coils (Siemens, Erlangen/Germany) for signal detection. A stack of 15 slices assured coverage of the whole LV. Late enhancement (LE) was obtained 10–15 minutes after the injection of gadopentetate dimeglumine 0.2 mmol/kg using an inversion recovery 2D turbo-gradient echo sequence.

### Reproducibility

Reproducibility of LSRsys and LSsys was assessed by repeated measurements in the same recordings. Intraobserver variation was assessed by repeated analysis of 30 studied subjects (15 patients with AL amyloidosis, 15 normal controls) and blinded to the initial results by one investigator (DL). Interobserver variation was done on the same datasets by two observers (DL and KH). Reproducibility was assessed using Bland and Altman analysis.

### Data Analysis

Data are presented as mean±standard deviation (SD) or median (quartiles), as appropriate. Differences on continuous data among 3 groups were compared using one-way analysis of variance (ANOVA) followed by either Tukeýs or Games-Howell multiple comparison post hoc tests as appropriate. Serum level of NT-proBNP and Troponin T showed a significant skewed distribution, difference between groups was compared using the Mann-Whitney U-statistic test. Multiple linear regression analysis was performed to identify predictors for the reduction of LSsys. Survival curves were calculated by the Kaplan-Meier method, and compared by Mantel-Cox log-rank tests. The end point was date of death or heart transplantation during follow-up. The mortality hazard ratios (HR) were calculated using univariate proportional-hazards regression analysis. The major determinants of mortality were identified by multivariate Cox proportional-hazards regression model after adjustment for age and gender. A *P* value <0.05 was considered statistically significant. Statistical analysis was performed using IBM SPSS, version 19 for Windows.

## Results

### Clinical Characteristics and Standard Echocardiography

Clinical features and the proportions of patients undergoing specific treatments for AL amyloidosis were shown in table 1. High-dose melphalan plus autologous stem-cell transplantation regimen was more frequently used while oral melphalan plus prednisone regimen was less frequently applied in compensated group than in decompensated group. Cardiac associated clinical data and standard echocardiographic data of the cohort are presented in table 2, specific echocardiographic and electrocardiographic parameters were shown in table 3. Serum NT-proBNP was available in 20 patients [median (quartiles), compensated: 1338 (562–35000) pg/mL; decompensated: 8765 (4610–17804) pg/mL; *P*>0.05]. Troponin T was available in 23 patients (compensated: 0.03 (0.01–0.07) ng/mL; decompensated: 0.12 (0.05–0.20) ng/mL; *P*>0.05). Eight patients received digitalis therapy for the purpose of rate control and/or for heart failure therapy. Thicker LV walls, smaller LV cavities, and enlarged left atria were present in patients compared to controls. EF and FS were similar between the controls and compensated patients but significantly reduced in decompensated patients. MAD_sept, MAD_lat, and TAPSE were significantly reduced in both patient groups. Table 3 showed that the proportion of pericardial effusion and advanced diastolic dysfunction were significantly higher in decompensated group than those in compensated group (all *P*<0.05).

**Table 1:** AL amyloidosis related clinical features and therapy responses.

	All patients	Compensated group	Decompensated group
	n = 33	n = 14	n = 19
Male (%)	58	64	53
Age (years)	65±10	64±8	66±11
AL amyloidosis (%)	64	57	68
AL amyloidosis plus multiple myeloma (%)	36	43	32
Light chain type			
κ light chain (%)	48	50	47
λ light chain (%)	52	50	53
Number of organ involvements	1.7±0.8	1.5±0.8	1.9±0.7
Renal (%)	64	57	68
Hepatic/gastrointestinal (%)	73	64	79
Lung (%)	9	7	11
Neuropathic (%)	6	14	0
Soft tissues/bone (%)	22	7	33
Treatment for AL amyloidosis (%)			
High-dose melphalan plus ASCT	42	64	26*
Oral melphalan or plus prednisone or bortezomib	33	14	47*
Lenalidomide plus Dex	9	7	10
Cyclophosphamid/CD/CAD/CTD	21	21	21
VCD/VTD/VMD	36	50	26
R-CVP	6	0	10
Various (VAD, immunotherapy, etc.)	9	14	5
Hematological response to treatment (%)	30	43	21

* *P*<0.05 vs. Compensated group. ASCT: autologous stem-cell transplantation; Dex: dexamethasone; CD: cyclophosphamide/dexamethasone; CAD: cyclophosphamide/adriamycin/dexamethasone; CTD: cyclophosphamide/thalidomide/dexamethasone; VCD: velcade/cyclophosphamide/dexamethasone; VTD: velcade/thalidomide/dexamethasone; VMD: velcade/melphalan/dexamethasone; R-CVP: rituximab plus/vincristine/prednisone; VAD: vincristine/adriamycin/dexamethasone.

**Table 2:** Cardiac related clinical data and standard echocardiographic characteristics according to clinical staging.

	Controls	Compensated group	Decompensated group
	n = 30	n = 18	n = 26
Male (%)	60	61	54
Age (years)	61±9	66±10	65±11
BMI (kg/m^2^)	24.6±3.0	23.4±3.0	24.4±3.4
Heart rate (beats/min)	69±10	73±8	84±12*
Systolic blood pressure (mmHg)	132±11	126±18	112±22
Diastolic blood pressure (mmHg)	82±9	74±11	71±13
Mean NYHA class	1±0	1.5±0.3*	3.1±0.4*†
Medication			
Digitalis	-	2 (11%)	6 (23%)
Angiotensin converting enzyme inhibitor	-	8 (44%)	8 (31%)
Angiotensin-II receptor typy-1 blocker	-	2 (11%)	5 (19%)
Aldosterone inhibitor	-	1 (6%)	5 (19%)
Beta blocker	-	6 (33%)	10 (39%)
LV end-diastolic dimension (mm)	50±4	43±7*	44±8*
LV mean thickness (mm)	9±1	13±3*	15±4*†
LA diameter (mm)	35±3	40±9*	44±8*
RV end-diastolic dimension (mm)	34±5	35±6	36±6
RA area (cm^2^)	15±3	17±5	21±6*†
RV free wall thickness (mm)	4±1	5±1*	6±1*†
Interatrial septum thickness (mm)	4±1	5±1	6±2*†
LV mass index (g/m^2^)	85±15	127±49	156±59*
LV stroke volume (ml)	78±19	51±19*	46±16*
LV fractional shortening (%)	37±7	34±7	25±8*†
LV ejection fraction (%)	66±6	63±7	52±12*†
Septal mitral annular displacement (mm)	12±1	7±3*	5±3*†
Lateral mitral annular displacement (mm)	14±2	10±3*	7±3*†
TAPSE (mm)	23±3	16±5*	13±4*†
E/A	1.1±0.3	1.2±0.7	1.8±0.9*†
E/E’	10±3	17±9*	25±10*†
DT (ms)	219±44	188±55	148±48*†

* *P*<0.05 vs. Controls; † *P*<0.05 vs. Compensated group. BMI: body mass index; NYHA: New York Heart Association; LV: left ventricle; LA: left atrial; RV: right ventricular; RA: right atrial; TAPSE: tricuspid annular plane systolic excursion; E/A: early diastolic filling velocity (E) to late diastolic filling velocity (A) ratio; E/E’: mitral inflow velocity (E) to tissue Doppler E’ ratio; DT: deceleration time of early filling.

**Table 3:** Echocardiographic and electrocardiographic parameters relevant to cardiac involvement.

	Control	All patients	Compensated group	Decompensated group	*P* value
	n = 30	n = 44	n = 18	n = 26	
Sparkling texture in the myocardium	0	35 (80%)	13 (72%)	22 (85%)	0.316
Pericardial effusion	0	21 (48%)	4 (22%)	17 (65%)	0.005
Enlarged left and right atria (LA diameter>40 mm, RA area>20 cm^2^)	0	18 (41%)	5 (28%)	13 (50%)	0.140
Diastolic pseudonormal or restrictive filling pattern	0	16/33 (48%)	3/15 (20%)	13/18 (72%)	0.003
Unexplained low voltage	0	21 (48%)	8 (44%)	13 (50%)	0.717
QRS-T wave pseudo-infarct changes	0	23 (52%)	9 (50%)	14 (54%)	0.802
I/II° atrioventricular block or/and left/right bundle branch block	0	35 (80%)	15 (83%)	20 (77%)	0.604
Atrial fibrillation	0	11 (25%)	3 (17%)	8 (31%)	0.288

LA: left atrium; RA: right atrium.

### Cardiac Magnetic Resonance Data

**Figure 1:**
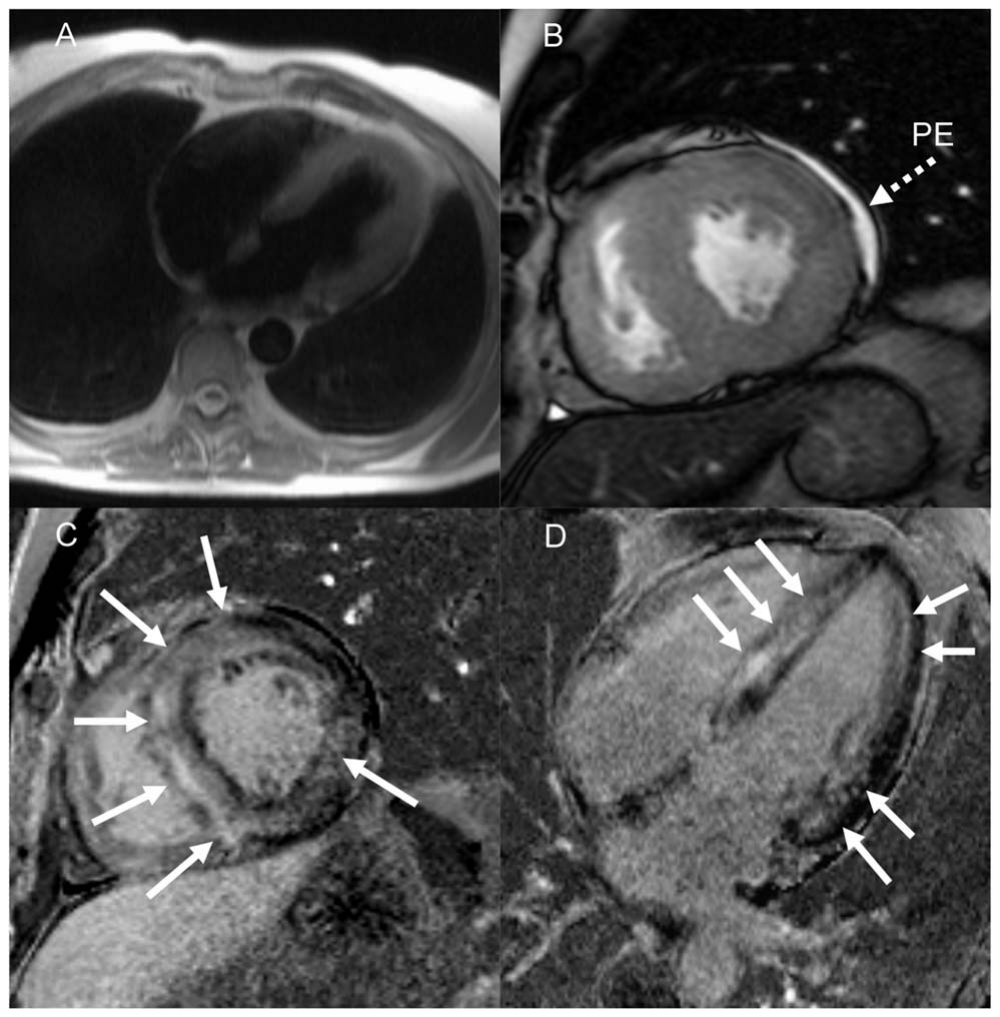
Cardiac magnetic resonance imaging in a patient with AL amyloidosis and LV hypertrophy. A (transverse T2 haste image) and B (short axis cine image) demonstrate left ventricular hypertrophy and minor pericardial effusion (dash arrow). C and D show late gadolinium enhancement images (short axis and horizontal long axis) presenting diffuse LE (solid arrows) in the left ventricular walls. LE: late enhancement; PE: pericardial effusion.

LE, a possible sign of myocardial interstitial deposition of amyloid fibrils, was detected by cMRI in 17 patients. Diffuse LE was documented in 11 patients and equally distributed in compensated (n = 6) and decompensated (n = 5) patients, localized (anteroseptal) LE was documented in 1 decompensated patient. Figure 1 shows example of diffuse LE in a patient.

LV longitudinal strain rate and strain data are shown in tables 4 and 5. LSRsys and LSsys were similar among groups in apical segments and significantly reduced in both patient groups in basal segments. LSsys at the mid segment was significantly reduced in all walls of the decompensated group. In general, LSRsys and LSsys at mid and basal segments were significantly lower in the decompensated group compared to the compensated group.

**Table 4:** Longitudinal peak systolic strain rate (LSRsys, s^−1^).

		Controls	Compensated group	Decompensated group
		n = 30	n = 18	n = 26
Septum	Apical	−1.26±0.34	−1.30±0.48	−1.18±0.47
	Mid	−1.02±0.22‡	−0.80±0.27*‡	−0.59±0.31*†‡
	Basal	−0.94±0.19‡	−0.60±0.24*‡	−0.41±0.25*†‡
Lateral wall	Apical	−1.24±0.27	−1.27±0.44	−1.15±0.42
	Mid	−1.08±0.31	−1.00±0.35	−0.68±0.29*†‡
	Basal	−1.20±0.26	−1.01±0.52	−0.49±0.31*†‡
Global LSRsys of the 6 segments in the 4−chamber view		−0.96±0.13	−0.94±0.29	−0.68±0.28*†
Inferior wall	Apical	−1.30±0.40	−0.99±0.37	−1.00±0.52*
	Mid	−1.10±0.28‡	−0.86±0.28*	−0.61±0.34*†‡
	Basal	−1.22±0.21	−0.94±0.32*	−0.66±0.31*†
Anterior wall	Apical	−1.24±0.51	−0.95±0.46	−1.28±0.52
	Mid	−1.11±0.36	−0.82±0.34*	−0.81±0.38*‡
	Basal	−1.16±0.40	−0.78±0.41*	−0.58±0.29*‡
Global LSRsys of the 6 segments in the 2−chamber view		−1.06±0.30	−0.80±0.27*	−0.69±0.29*
Posterior wall	Apical	−1.21±0.33	−1.13±0.37	−0.98±0.42
	Mid	−1.17±0.31	−0.96±0.29	−0.62±0.33*†‡
	Basal	−1.35±0.27	−0.92±0.33*	−0.64±0.29*†‡
Anteroseptal wall	Apical	−1.28±0.37	−1.44±0.53	−1.27±0.51
	Mid	−1.09±0.23‡	−0.92±0.37‡	−0.96±0.42‡
	Basal	−0.92±0.14‡	−0.54±0.27*‡	−0.50±0.30*‡§
Global LSRsys of the 6 segments in the apical long axis view		−1.04±0.16	−0.92±0.20	−0.72±0.28*†

* *P*<0.05 vs. Controls; † *P*<0.05 vs. Compensated group; ‡ *P*<0.05 vs. apical; § *P*<0.05 vs. Mid.

**Table 5:** Longitudinal, circumferential, and radial peak systolic strain (%).

		Controls	Compensated group	Decompensated group
		n = 30	n = 18	n = 26
Longitudinal systolic strain (LSsys, %)				
Septum	Apical	−21.2±4.8	−19.7±7.1	−15.5±4.9*†
	Mid	−18.2±3.6‡	−13.0±5.3*‡	−8.0±3.9*†‡
	Basal	−16.4±2.7‡	−9.3±5.3*‡	−5.4±3.1*†‡
Lateral wall	Apical	−19.1±4.3	−18.7±6.2	−14.8±5.7*
	Mid	−17.9±3.4	−13.6±5.5*	−8.6±4.6*†‡
	Basal	−18.8±3.9	−11.5±6.8*‡	−5.2±4.0*†‡
Global LSsys of the 6 segments in the 4−chamber view		−18.3±2.0	−14.1±5.1*	−9.4±3.7*†
Inferior wall	Apical	−21.0±4.7	−14.8±6.7*	−13.0±5.8*
	Mid	−19.3±3.7	−11.4±4.4*	−8.6±5.1*‡
	Basal	−19.3±3.0	−10.9±4.5*	−7.4±3.9*†‡
Anterior wall	Apical	−19.4±5.6	−14.6±7.1*	−14.6±5.3*
	Mid	−18.6±4.6	−12.3±6.2*	−10.2±3.8*‡
	Basal	−18.7±4.8	−10.0±6.3*	−7.6±4.1*‡
Global LSsys of the 6 segments in the 2−chamber view		−19.0±3.0	−12.3±4.7*	−9.9±3.5*
Posterior wall	Apical	−21.0±4.1	−16.9±6.1	−12.7±6.0*
	Mid	−19.2±4.0	−12.3±5.5*	−7.3±5.1*†‡
	Basal	−20.0±4.0	−9.8±6.8*‡	−6.3±4.0*‡
Anteroseptal wall	Apical	−22.1±6.0	−19.7±5.7	−16.0±6.4*
	Mid	−19.9±4.1	−14.3±5.4*	−12.3±5.6*
	Basal	−15.9±2.9‡§	−8.2±5.0*‡§	−7.9±5.0*‡§
Global LSsys of the 6 segments in the apical long axis view		−19.4±2.4	−13.2±4.3*	−9.7±4.4*†
Circumferential systolic strain (CSsys, %)				
Anteroseptal wall		−25.2±4.9	−20.6±6.4	−15.4±8.2*
Anterior wall		−22.7±4.3	−17.9±5.3	−12.8±7.7*†
Lateral wall		−16.4±4.5	−11.2±3.7*	−8.4±5.4*
Posterior wall		−14.0±6.4	−8.5±5.2*	−7.9±4.7*
Inferior wall		−15.4±5.5	−11.7±8.4	−10.9±4.4*
Septum		−20.7±3.9	−17.3±7.1	−14.0±6.2*
Global		−18.0±2.5	−13.8±4.5*	−11.3±5.4*
Radial systolic strain (RSsys, %)				
Anteroseptal wall		39.2±19.1	36.2±17.2	17.3±11.1*†
Anterior wall		46.2±19.0	40.5±19.3	20.8±13.7*†
Lateral wall		52.5±20.4	44.0±19.7	22.7±15.5*†
Posterior wall		54.7±19.9	43.5±18.3	22.2±14.5*†
Inferior wall		51.2±18.4	39.4±16.0	19.5±11.6*†
Septum		42.7±19.9	35.1±14.6	16.5±9.3*†

* *P*<0.05 vs. Controls; † *P*<0.05 vs. Compensated group; ‡ *P*<0.05 vs. Apical; § *P*<0.05 vs. Mid.

Global circumferential systolic strain was significantly reduced in both patient groups. Radial Ssys in all 6 walls was similar between controls and the compensated group, but was significantly reduced in the decompensated group (table 5).

### Base-to-apex Deformation Gradient

**Figure 2:**
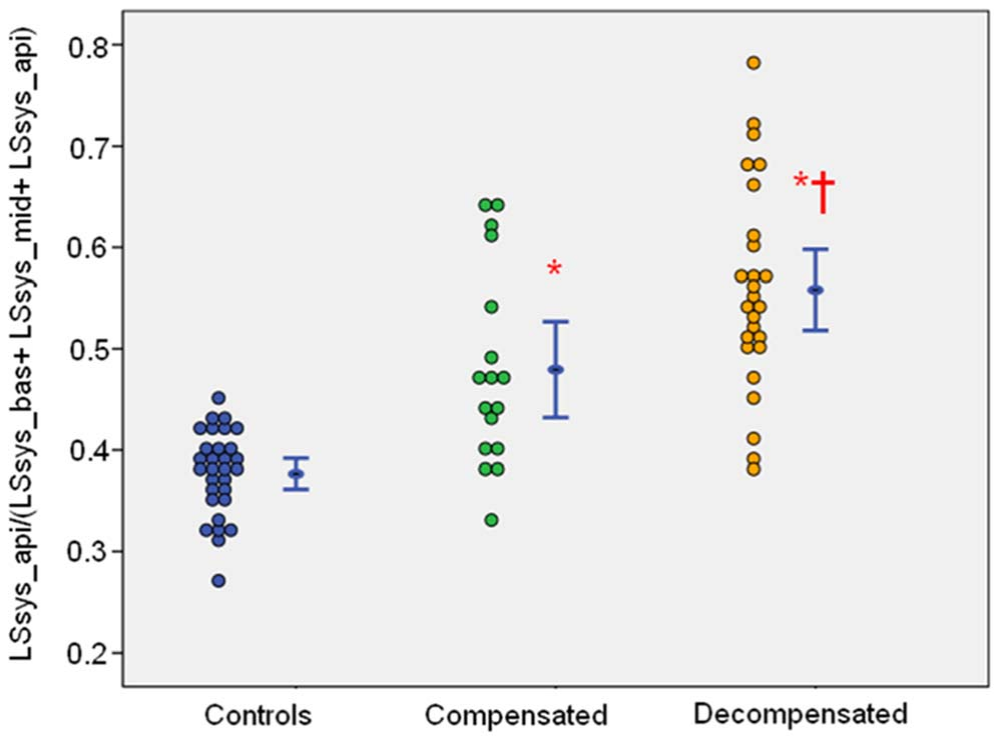
Scatterplot and error bar (mean±2SD) of LSsys_api/(LSsys_bas+LSsys_mid+LSsys_api) in the septum. Pathological base-to-apex gradient was defined as LSsys_api/(LSsys_bas+LSsys_mid+LSsys_api) ≥0.45. Pathological gradient of the septum was present in 32 out of 44 patients (73%) with AL amyloidosis and LV hypertrophy [9 (50%) compensated, 23 (88%) decompensated, P<0.05]. *: P<0.05 vs. Controls; †: P<0.05 vs. Compensated group. LSsys_api: apical longitudinal systolic strain; LSsys_mid: mid longitudinal systolic strain; LSsys_bas: basal longitudinal systolic strain.

There was a base-to-apex deformation gradient with higher apical LSRsys and LSsys values and lower mid and basal LSRsys and LSsys values in both patient groups as well as in the controls. Accordingly, we established a ratio to quantify this base-to-apex gradient. The ratio was obtained by dividing apical LSsys with the sum of basal, mid, and apical LSsys (LSsys_api/(LSsys_bas+LSsys_mid+LSsys_api)), ratio≥0.45 (0.45 represents the maximal ratio of the control group) indicates pathological gradient. The prevalence of pathological gradient in the septum was 73% (32/44) in all patients [9 (50%) compensated patients, 23 (88%) decompensated patients, *P*<0.05] (figure 2).

### Echocardiographic Staging

**Figure 3:**
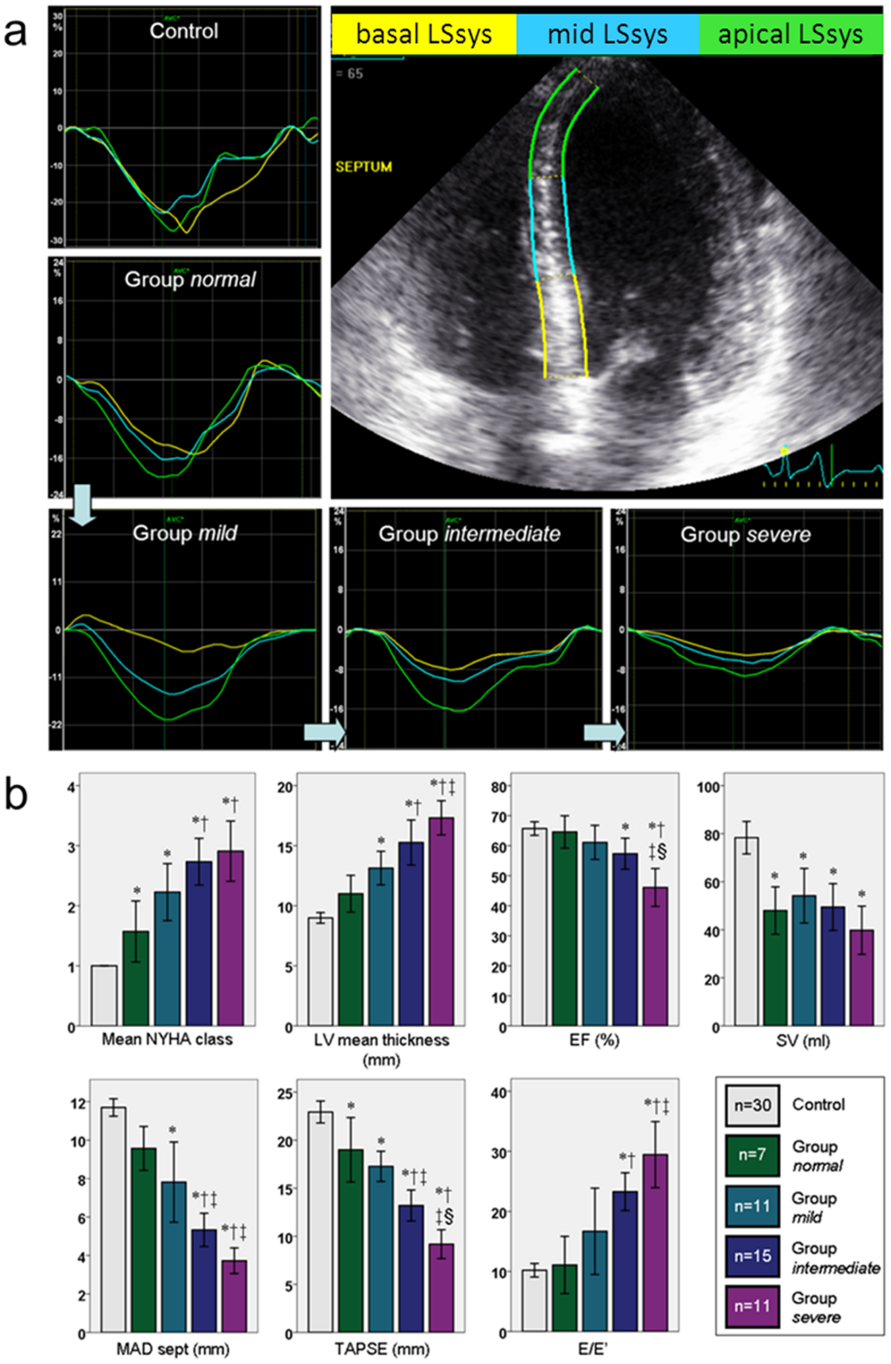
Echocardiographic staging and standard echocardiographic parameters among 4 subgroups. a: Reduced LSsys was defined as less than the mean from the control group minus 2SD. Group *normal*: no reduction of LSsys in any segment; Group *mild*: only basal reduced LSsys; Group *intermediate*: basal and mid reduced LSsys; Group *severe*: reduced LSsys in all apical, mid and basal segments. b: Mean NYHA class, LV mean thickness and E/É increased while left ventricular ejection fraction, stroke volume, septal mitral annular displacement, as well as tricuspid plane annular systolic excursion decreased in proportion to increased number of involved segments with reduced LSsys.

Applying the knowledge of reduced septal LSsys in the decompensated patients we staged the entire cohort according to the presence or absence of reduced LSsys at apical, mid, and basal segments in the septum (figure 3a).

Cutoff value of reduced LSsys was calculated as mean minus 2SD in the control group (11.0% at basal, 11.0% at mid and 11.6% at apical segment) [14]. Group *normal:* no reduction of LSsys in any segment; group *mild*: only basal reduced LSsys; group *intermediate*: basal and mid reduced LSsys and group *severe*: reduced LSsys in all apical, mid and basal segments. The comparisons of the clinical and standard echocardiographic parameters among subgroups based on echocardiographic staging were listed in table 6. A gradually increase in mean NYHA class, mean LV thickness, LAD, RAA, IASd and E/E? and a gradually decreasing trend in FS, EF, SV, MAD_sept, MAD_lat, and TAPSE were seen from group *mild* to group *severe* (table 6 and figure 3b).

**Table 6:** Clinical and standard echocardiographic characteristics according to echocardiographic staging.

	Normal	Mild	Intermediate	Severe	*P* value
	n = 7	n = 11	n = 15	n = 11	
Male (%)	43	54	53	73	0.617
Age (years)	60±8	68±7	63±11	70±11	0.060
BMI (kg/m^2^)	23±2	25±4	24±3	23±3	0.539
Heart rate (beats/min)	78±12	81±11	78±13	81±11	0.904
Systolic blood pressure (mmHg)	119±16	131±22	113±19	110±22	0.127
Diastolic blood pressure (mmHg)	74±10	74±14	71±12	71±14	0.911
Mean NYHA class	1.6±0.7	2.2±0.8	2.7±0.7*	2.9±0.8*	0.004
Chemotherapy	6 (86%)	10 (91%)	7 (47%)	4 (36%)	0.138
Stem cell transplantation	5 (71%)	4 (36%)	4 (27%)	1 (9%)	0.083
Medication					
Diuretics	4 (57%)	8 (73%)	9 (60%)	9 (82%)	0.189
Digitalis	0	2 (18%)	4 (27%)	2 (18%)	0.478
Angiotensin converting enzyme inhibitor	3 (43%)	4 (36%)	6 (40%)	3 (27%)	0.963
Angiotensin-II receptor typy-1 blocker	0	5 (45%)	0	2(18%)	0.013
Aldosterone inhibitor	0	0	4 (27%)	2 (18%)	0.128
Beta blocker	2 (29%)	5 (45%)	6 (40%)	3 (27%)	0.868
LV end-diastolic dimension (mm)	42±7	44±7	42±7	45±7	0.610
LV mean thickness (mm)	11±2	13±2	15±4*	17±2*†	<0.001
LA diameter (mm)	35±8	39±8	44±9	47±4*†	0.010
RV end-diastolic dimension (mm)	33±6	35±6	33±5	39±5	0.075
RA area (cm^2^)	15±3	17±7	20±6	24±4*	0.016
RV free wall thickness (mm)	5±1	5±1	6±1	7±1*†‡	0.010
Interatrial septum thickness (mm)	5±1	5±1	6±2	7±1*†‡	0.046
LV mass index (g/m^2^)	91±24	127±34	145±57	198±46*†‡	<0.001
LV stroke volume (ml)	48±13	54±19	49±19	40±17	0.291
LV fractional shortening (%)	34±5	32±8	28±8	21±8*†	0.003
LV ejection fraction (%)	64±7	61±9	57±10	46±10*†‡	0.001
Septal mitral annular displacement (mm)	9±1	8±3	5±2*	4±1*†‡	<0.001
Lateral mitral annular displacement (mm)	12±2	10±3	7±2*	5±1*†‡	<0.001
TAPSE (mm)	19±4	17±3	13±3*†	9±2*†‡	<0.001
E/A	1.3±0.8	1.0±0.5	1.5±0.9	2.2±0.7*†	0.040
E/E’	11±6	17±11	23±5*	29±9*†	<0.001
DT (ms)	194±68	180±41	178±66	128±35*†‡	0.049

* *P*<0.05 vs. Normal; † *P*<0.05 vs. Mild; ‡ *P*<0.05 vs. Intermediate. For abbreviations, see table 2.

Multiple linear regression analysis showed that LV wall thickness (*P* = 0.006), EF (*P* = 0.049), and TAPSE (*P* = 0.007) were significantly associated with echocardiographic staging (*normal*, *mild*, *intermediate* and *severe*) while NYHA functional class (*P* = 0.165) and E/É (*P* = 0.073) were not associated with echocardiographic staging in AL amyloidosis patients of this cohort. The multiple linear regression equation is as follows:

Echo staging = −1.205+0.204×NYHA class+0.449×LV wall thickness (mm)+0.326×EF (%)+0.450×TAPSE (mm)+0.353×E/E? (r = 0.845; r^2^ = 0.715; *P*<0.001; standard error of the estimate = 0.616)

### Follow-up

**Figure 4:**
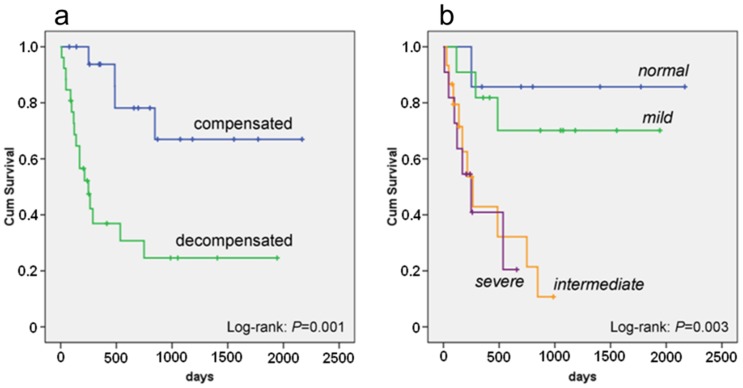
Kaplan-Meier plots comparing survival of patients with compensated and decompensated patient group and with echocardiographic staging group *normal, mild, intermediate, and severe*. Figure 4a shows that survival was significantly reduced in the decompensated patient group (35% vs. compensated 78%, P = 0.001). Figure 4b shows that reduced LSsys at mid and apical segments (group intermediate and severe) strongly predicts reduced survival in AL amyloidosis patients with cardiac involvement (P = 0.003).

The 44 patients were followed up for a median of 345 days (quartiles: 141–846 days), 20 patients (45%) died and 1 patient (2%) underwent heart transplantation. The Kaplan-Meier survival analysis (figure 4) showed survival probability was significantly reduced in the decompensated group (35% vs. compensated 78%, Mantel-Cox log-rank: *P* = 0.001). When looking at subgrouping by echocardiography the distribution of death or heart transplantation was 1/7 (14%) in the group *normal*, 3/11 (27%) in the group *mild*, 10/15 (67%) in the group *intermediate*, and 7/11 (64%) in the group *severe* (*P* = 0.003). Cox proportional-hazards regression analysis showed that patients with reduced mid-septum LSsys had worse survival (35%) than patients with preserved LSsys (78%, *P* = 0.005). Mid-septum LSsys <11% suggested a 4.8-fold mortality risk as compared to those with mid-septum LSsys ≥11% during follow-up. However, there was no significant predictive information for mortality risk in either LV wall thickness or EF. Multivariate Cox proportional-hazards regression model showed NYHA class and the mid-septum LSsys were independent predictors for survival (table 7). Mortality was similar between patients with LE (5/12, 42%) and without LE (3/5, 60%, *P*>0.05). Regression analysis of AL amyloidosis related predictors on mortality showed that high-dose melphalan plus autologous stem-cell transplantation, oral melphalan or plus prednisone or bortezomib and number of involvement organs were predictors for increased risk of death for AL amyloidosis patients with cardiac involvement (table 8).

**Table 7:** Cox proportional-hazards regression analysis of clinical and echocardiographic predictors on mortality.

	Wald	Hazard ratio	95% CI	*P* value
Univariate analysis				
Age	0.156	1.01	0.97 – 1.05	0.693
Gender	0.553	1.50	0.52 – 4.32	0.457
NYHA class>2	2.508	2.77	0.79 – 9.78	0.113
LV mean thickness≥14 mm	0.003	1.03	0.31 – 3.49	0.959
Ejection fraction<50%	0.844	1.80	0.52 – 6.26	0.358
Mid-septum LSsys<11%	5.600	4.80	1.31 – 17.56	0.018
Multivariate analysis				
NYHA class	3.995	3.21	1.02 – 10.06	0.046
Mid-septum LSsys (%)	6.516	4.54	1.42 – 14.52	0.011

CI: confidence interval; NYHA: New York Heart Association; LV: left ventricular; LSsys: longitudinal systolic strain.

**Table 8:** Cox proportional-hazards regression analysis of AL amyloidosis related predictors on mortality.

	Wald	Hazard ratio	95% CI	*P* value
Univariate analysis				
Age	0.050	0.99	0.91–1.07	0.823
Gender	0.356	0.83	0.45–1.53	0.551
Light chain type	0.151	1.26	0.39–4.06	0.698
Number of involvement organs	8.714	4.07	1.60–10.33	0.003
Hematological response to treatment	0.035	0.88	0.23–3.40	0.851
High-dose melphalan plus ASCT	5.182	6.58	1.30–33.35	0.023
Oral melphalan or plus prednisone or bortezomib	6.656	13.22	1.86–94.03	0.010
Multivariate analysis				
High-dose melphalan plus ASCT	5.118	6.35	1.28–31.48	0.024
Oral melphalan or plus prednisone or bortezomib	8.082	11.22	2.12–59.42	0.004
Number of involvement organs	8.854	3.549	1.54–8.18	0.003

CI: confidence interval; ASCT: autologous stem-cell transplantation.

### Reproducibility

The intraobserver and interobserver variability was assessed in 480 measured segments. The intraobserver absolute bias were 0.01s^−1^ (−0.01–0.04) and 0.5% (0.3–0.8) for LSRsys and LSsys respectively. The interobserver absolute bias were 0.04s^−1^ (0.01–0.07) and 0.4% (0.05–0.7) for LSRsys and LSsys respectively.

## Discussion

This study comprehensively assessed the cardiac function of patients with CA by analysis of clinical, standard echocardiography and STI-derived regional myocardial function data. The main findings of the present study are: 1) longitudinal function is reduced whereas radial function remains largely preserved in these patients; 2) there is an intra-wall longitudinal deformation gradient due to preserved LSsys at apical segments and significantly reduced LSsys at mid and basal segments; 3) The intra-wall longitudinal deformation gradient and the number of affected segments with reduced LSsys could be used for staging of patients with CA and an increased number of segments with reduced LSsys is linked with advanced clinical stage and poorer outcome.

### Myocardial Function in AL Amyloidosis

The current study demonstrated that reduced longitudinal but preserved radial function could be already detected in these patients in the absence of global marker (EF) changes. In line with previous studies [15], [16], we showed that EF, the routine clinical parameter assessing LV global systolic function, was preserved in these patients up to the decompensated stage. This suggests that evaluating deformation parameter is superior to EF for staging these patients since EF (calculated using the Simpson formula) which represents the volume change resulting from all deformation components, does not discriminate between circumferential and longitudinal function [17], [18].

Serial clinical studies suggested that myocardial deformation imaging (i.e., strain rate imaging) could be used to detect more subtle regional myocardial motion and deformation changes and thus reliably reveals cardiac impairment in systemic amyloidosis patients with cardiac impairments [12], [19], [20]. In a previous study, Sun et al. reported that the longitudinal, radial and circumferential strain detected by 2-dimensional strain echocardiography were all significantly lower in patients with cardiac amyloidosis compared to healthy controls but also to subjects with LV hypertrophy caused by hypertrophic cardiomyopathy or hypertensive heart disease, and cardiac amyloidosis patients was differentiated from the other hypertrophic groups by longitudinal strain<12% [21]. The current study demonstrated that patients with CA exhibited a pronounced intra-wall base-to-apex gradient due to almost absent long axis deformation at basal segments and preserved regional deformation at apical segments. We were able to show that this gradient could be used for staging the disease progression and reveal relevant prognostic information in these patients. The functionality at apical segments also became compromised at the very late course of the disease, hence, patients with reduced apical deformation had the worst clinical status( = high NYHA class+congestive heart failure), the most pronounced remodeling( = LV hypertrophy), and the worst global LV systolic( = EF), diastolic( = E/É) and RV function( = TAPSE). In a prognostic study with 119 AL amyloidosis patients, Koyama et al. showed that the mean basal strain was a powerful predictor of clinical outcome [22]. Whereas, our findings suggested that mid septal LSsys but not basal or apical septal LSsys was a more important prognostic indicator and mid-segment involvement (LSsys_mid<11.0%) was linked with prognosis deterioration with a mortality risk of 65% in about 9 months. In this cohort, the reduction of basal septal LSsys was presented in the majority (37/44, 84%) of the patients, hence, our results hinted that basal septal LSsys might not be a sensitive prognostic indicator for outcome in patients with CA.

The underlying pathomechanisms responsible for the early functional reduction at basal segments in these patients are speculative. Brenner et al. reported that human light chain proteins could directly impair active force through an increase in cellular oxidant stress [23]. This directly toxic phenomenon should, however, homogeneously impact on all LV segments. Additionally, amyloid deposition and tissue damage detected by cMRI [24], seems to predominantly occur at the endo- and epi-cardial sides, where the longitudinally oriented fibers are located. Thus, long axis function might be impaired at the early disease course. In theory, radial function should also decreases if both longitudinal and circumferential deformation deteriorates. However, in compensated group, we observed preserved radial deformation while the other components decreased. This can be explained by the fact that these hearts showed significantly increased ventricular mass together with decreased cavity volumes. Even with decreased longitudinal and (slightly) decreased circumferential deformation, the thicker tissue with a smaller diameter retains its total tissue volume and, capacities of thickening. Additionally, it is known that loading conditions are not homogeneously distributed throughout the LV, with the highest wall stress at the basal septal segments because of the non-spherical left ventricle geometry and the largest local radius of the ventricular curvature [25], [26]. This greater local wall stress might be the reason for the well-known apoptosis, collagen formation and subsequent fibrosis in these segments [27]. Taken together, these aforementioned unfavorable effects, increased wall stress and changes in tissue elasticity because of fibrosis comprehensively might consequently result in observed regional myocardial deformation abnormalities in these patients. Moreover, locally increased turbulent flow in the left ventricular outflow tract near the basal segment might also aggravate apoptosis [27] and subsequent fibrosis and thus contribute to reduced contractility at basal segments in these patients with CA.

In fact, the observed reduction in longitudinal strain in the basal and mid segments with a preserved strain in apical segments was also observed in patients with decompensated hypertrophic cardiomyopathy [28], future studies are warranted to explore if the observed "baso-apical" strain gradient is a special "pathognomonic feature" or not for patients with cardiac amyloidosis.

### Prognostic Implication

Previous studies have demonstrated that LV hypertrophy identifies a population at high risk for cardiovascular disease and predicts an increased risk of cardiovascular morbidity and death independent of age, blood pressure, cigarette use, diabetes, obesity [29], [30]. It has also been suggested that LV hypertrophy and reduced EF are associated with poor outcome in AL cardiac amyloidosis patients [3], [31]. The current study shows combining conventional echocardiographic parameters with the STI derived base-to-apex intra-wall longitudinal deformation gradient is helpful for staging the patients with CA, and deformation changes is superior to hypertrophy and EF on predicting the prognosis in patients with CA.

### Study Limitations

The patient cohort is relatively small in the present study. Studies with larger patient number are warranted to overcome this limitation and verify the outcome results. The prognostic potential of NT-proBNP and troponin in patients with AL amyloidosis is widely accepted. However, NT-proBNP and troponin were available in only 23 patients in our cohort. It is therefore very difficult to determine the prognostic value of these cardiac biomarkers due to the small population in this cohort. In this cohort, chemotherapy was applied in 60% of the patients prior to first echocardiography and chemotherapy was added further in 34% of the patients during follow-up. Since the hematological response to treatment and time-to-response are important predictors of survival in patients with systemic light-chain amyloidosis [32], [33], the results bias due to various timing of chemotherapy in this cohort should be considered.

## Conclusion

Besides the traditional parameters indicating cardiac involvement, the assessment of regional myocardial deformation by 2D-STI provides important information on cardiac function and staging for patients with CA. The longitudinal intra-wall base-to-apex deformation gradient is helpful to detect cardiac impairments in the absence of reduced EF value. An increasing number of segments with reduced longitudinal systolic strain is linked with advanced clinical stage and poorer outcome in patients with CA.
